# A CMOS SPAD Imager with Collision Detection and 128 Dynamically Reallocating TDCs for Single-Photon Counting and 3D Time-of-Flight Imaging

**DOI:** 10.3390/s18114016

**Published:** 2018-11-17

**Authors:** Chao Zhang, Scott Lindner, Ivan Michel Antolovic, Martin Wolf, Edoardo Charbon

**Affiliations:** 1 Quantum and Computer Engineering, Delft University of Technology, Mekelweg 4, 2628CD Delft, The Netherlands; 2 Biomedical Optics Research Laboratory, University of Zurich, Rämistrasse 71, 8006 Zürich, Switzerland; scott.lindner@epfl.ch (S.L.); martin.wolf@usz.ch (M.W.); 3 Advanced Quantum Architecture Laboratory, École Polytechnique Fédérale de Lausanne (EPFL), Route Cantonale, 1015 Lausanne, Switzerland; michel.antolovic@epfl.ch (I.M.A.); edoardo.charbon@epfl.ch (E.C.); 4 Kavli Institute of Nanoscience, 2628CJ Delft, The Netherlands

**Keywords:** single-photon avalanche diode, SPAD, time-of-flight, dynamic reallocation, time-to-digital converter, collision detection bus, image sensor, light detection and ranging, LiDAR

## Abstract

Per-pixel time-to-digital converter (TDC) architectures have been exploited by single-photon avalanche diode (SPAD) sensors to achieve high photon throughput, but at the expense of fill factor, pixel pitch and readout efficiency. In contrast, TDC sharing architecture usually features high fill factor at small pixel pitch and energy efficient event-driven readout. While the photon throughput is not necessarily lower than that of per-pixel TDC architectures, since the throughput is not only decided by the TDC number but also the readout bandwidth. In this paper, a SPAD sensor with 32 × 32 pixels fabricated with a 180 nm CMOS image sensor technology is presented, where dynamically reallocating TDCs were implemented to achieve the same photon throughput as that of per-pixel TDCs. Each 4 TDCs are shared by 32 pixels via a collision detection bus, which enables a fill factor of 28% with a pixel pitch of 28.5 μm. The TDCs were characterized, obtaining the peak-to-peak differential and integral non-linearity of −0.07/+0.08 LSB and −0.38/+0.75 LSB, respectively. The sensor was demonstrated in a scanning light-detection-and-ranging (LiDAR) system equipped with an ultra-low power laser, achieving depth imaging up to 10 m at 6 frames/s with a resolution of 64 × 64 with 50 lux background light.

## 1. Introduction

The demand for 3D imaging systems is growing rapidly, with applications such as facial recognition, robotics, bioimaging, and LiDAR. One of the widely used ranging approaches is triangulation, which, in current implementations, is limited in range due to the base-line parameters [1]. The time-of-flight (TOF) technique, on the other hand, measures the delay between the emitted and backscattered light directly (dTOF) or indirectly (iTOF) [2,3,4,5,6,7,8,9,10]. The iTOF is based on per-pixel photon-demodulators which achieves high resolution but over relatively short ranges, usually within 20 m [2,3,4]. Whilst dTOF has some key advantages in comparison with iTOF, e.g., longer range, and multi-echo detection [5,6,7,8,9,10]. The implementation of dTOF involves the capability of resolving the arrival time of reflected photons, which requires photodetectors with a high gain.

Single-photon avalanche diodes (SPADs) with the ability to produce digital pulse from a single detected photon and excellent timing response, have been designed for various applications, including TOF imaging. With the single-photon sensitivity, one SPAD can be coupled with a TDC, thus large format of pixel arrays can be designed to perform high spatial resolution 3D imaging [5,11,12]. However, with this per-pixel TDC architecture, the fill factor is limited due to the large area occupancy of the TDC circuitry, which reduces the photon detection efficiency. For instance, Ref. [11] reports a large SPAD array (160 × 128) with low fill factor (1%) in a large pixel pitch of 50 μm. On the other hand, in the readout stage, to avoid the data transmission of the pixel addresses, all the TDCs will be output sequentially regardless of data validity, which reduces the readout efficiency both in time and energy due to the null events communication. An event-driven readout method has been presented in [12], which skips the null events by pre-processing the data with a high speed pipeline before readout. Whereas the total output bandwidth of 42 Gbps was achieved at the cost of average power of 8.79 W, which meant the system required a liquid cooling system. In fact, due to the digitization of photons, a large volume of data can be generated then read off-chip via bandwidth limited IOs. In most situations, instead of the TDC number, the IO bandwidth is the major limitation to the photon throughput [5,8,11,12,13]. Therefore, to avoid the distortion due to photon pileup, the pixel activity must be restricted to 1–3%. In this case, only a small proportion of the TDCs can be triggered, which implies the per-pixel TDC architecture is not the optimal option in terms of the readout efficiency.

In this paper, we report on the design and characterization of a SPAD sensor featuring a TDC sharing architecture that performs dTOF imaging for low light level applications, e.g., fluorescence lifetime imaging microscopy (FLIM), indoor facial recognition. It features an array of 32 × 32 pixels, where each 32 pixels in one column share 4 TDCs via a timing line respectively. A collision detection bus is used to detect two or more SPAD events. TDCs are shared in a dynamic reallocation scheme to detect events sequentially. The TDC number is determined based on the analysis of the IO readout bandwidth to achieve the same photon throughput as that of per-pixel TDC architecture. The readout efficiency is improved with an event-driven readout method that only the valid events will be transmitted off-chip, thus enabling a photon throughput of 222 million counts per second (Mcps) and 465 Mcps in photon timestamping and photon counting modes, respectively.

The sensor was firstly demonstrated with flash imaging that achieved millimetric depth resolution. To extend the spatial resolution, the sensor was measured and characterized in a 2D scanning LiDAR system, equipped with a dual-axis galvanometer scanner and a low power 637 nm diode laser (with average and peak power of 2 mW and 500 mW, respectively). In this setup, all the pixels were combined as one component for TCSPC detection. Instead of transmitting every event to the computer, the histogram of each point was constructed in the field-programmable gate array (FPGA) and transmitted through a universal serial bus 3 (USB3) for final distance calculation and image display, thus reducing the required transmission bandwidth. Real-time and accurate range images were obtained with a resolution of 64 × 64 pixels, at 6 frames/s, and within a range of 10 m on a 40% reflectivity target. Distance measurements up to 50 m revealed 6.9 cm non-linearity and 0.62 cm precision.

Meanwhile, to improve the background light suppression, a new sensor architecture is proposed based on the collision detection bus. Coincidence detection circuits with smaller logic could be achieved to enable a large pixel array for high background light applications, such as automotive LiDAR.

The paper is organized as follows: the sensor architecture is described in detail in Section 2. Section 3 shows the sensor characterization and the experimental results. The proposed sensor architecture is discussed in Section 4. Finally, conclusions are drawn in Section 5.

## 2. Sensor Design

### 2.1. Sensor Architecture

**Figure 1:**
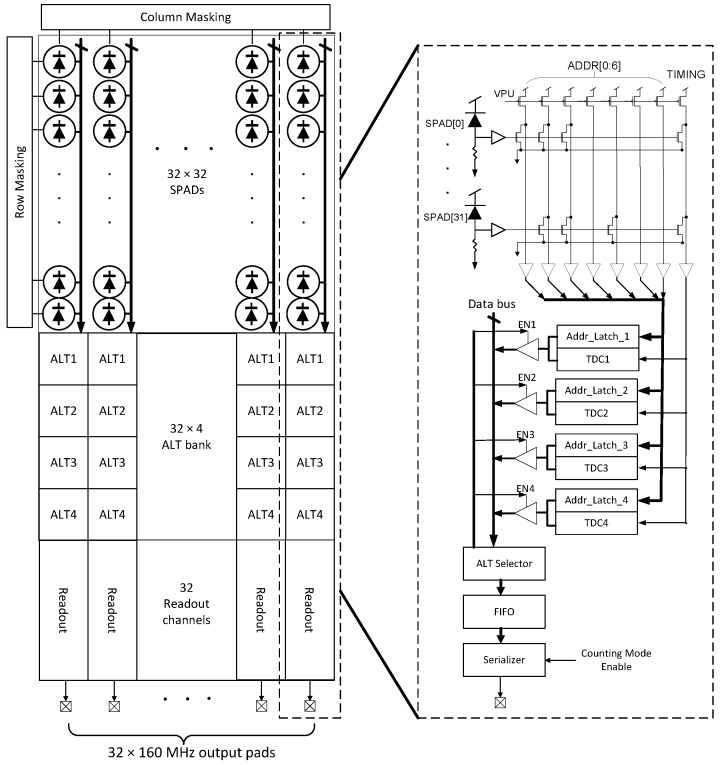
Image sensor architecture.

The block diagram of the sensor is illustrated in Figure 1, where the pixel, collision detection bus and address latching mechanism are also applied in [14], but with a different readout scheme and TDC architecture. The pixel array is connected to the timing circuitry with shared bus architecture [15,16]. In this instance, all 32 pixels in each column share a single address bus and a timing line. The pixel addresses are coded in a collision detection approach where collision events lead to an invalid address output, thus allowing collisions to be identified. Due to the TDC sharing architecture, each event occupies the bus for a set period, the bus dead time. To reduce this duration, a monostable circuitry is included in the pixel structure. Furthermore, the shared architecture also implies that very noisy SPADs could occupy the bus for large periods, thus reducing the sensitivity of the column to real photon arrivals. Therefore, a set of row and column masking shift registers were implemented to shut down noisy pixels according to the DCR level.

At the bottom of the columns, a bank of 128 address-latch-and-TDC (ALTDC) functional blocks are used to capture the pixel address and to measure photon arrival time. Instead of a fixed pixel to TDC connection architecture, a dynamically reallocating approach was implemented where events are distributed sequentially among 4 TDCs to improve the detection throughput. The TDCs employ an architecture based on a ring oscillator, the frequency of which is set via an external voltage. In this case, the TDC has a 12-bit range where the ring-oscillator is operated at 2.5 GHz to achieve a least-significant bit (LSB) of 50 ps.

Time-of-flight data is read from each column with a dedicated readout block, which serializes data and streams it off-chip via a column-wise 160 MHz data pad. Each one works in an event-driven readout approach such that only the ALTDCs, which have detected photons will be readout through a tri-state bus. The data is firstly pushed into a first-in-first-out (FIFO) block and then a serializer reads the events out in UART format. Compared with the frame based readout method in [11] that reads all the TDCs regardless of data validity and the power-hungry pipelined datapath readout method in [12], a higher energy efficiency is achieved with our approach. The readout can operate in either photon timestamping (PT) or photon counting (PC) modes. In PT mode, both the TOF information and pixel address is read out from the sensor. A transmitted event comprises 23 bits including 1-bit start flag, 2-bit TDC identification number, 12-bit TDC code, 7-bit address code, and 1-bit stop flag. While in PC mode, the sensor only transmits 1-bit start flag, 7-bit address code, and 1-bit stop flag that the data length is reduced to 11 bits. As such, a maximum photon throughput of 222 Mcps and 465 Mcps can be achieved in PT and PC mode, respectively.

### 2.2. Pixel Schematic and Collision Detection Bus

**Figure 2:**
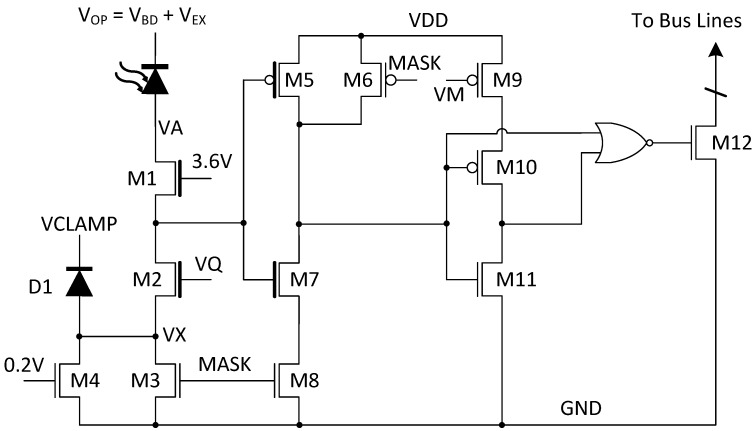
Pixel schematic with cascaded quenching.

The sensor employs a SPAD with a p-i-n structure reported in [17]. In order to achieve both high PDP and fill factor, a cascoded quenching circuit, Figure 2, is used to allow the SPAD to operate at excess bias voltages up to 5.2 V without exceeding the 3.6 V reliability limit across the gate-source, gate-drain and drain-source junctions of any device [18]. Since this technique only uses transistors, the layout is very dense, achieving an overall fill factor of 28% with a pixel pitch of 28.5 μm.

In the pixel, passive quenching and recharge is controlled by voltage VQ, which is typically biased at 0.8 V leading to a 50 ns dead time. Noisy pixels are disabled with transistors M3, M4 and M6. If voltage MASK is set as low, M3 operates in cut off region and the impedance is typically at the level of giga ohm, thus preventing the SPAD from recharging. However, the leakage current from SPAD may accumulate at the anode and increase the voltage of VA over the tolerant limit. Over time this could cause M1 to breakdown. To ensure the safety of the pixel, a parallel transistor M4, with its gate biased at 0.2 V, is used to provide a lower impedance path to drain out the leakage current and to prevent VA from increasing. Furthermore, a diode D1 clamps VX at a safe voltage VCLAMP, normally at 1.8 V, to protect M3 and M4 from high voltage in any case. A configurable monostable circuit comprising M9, M10, M11 and a NOR gate was implemented to reduce the pulse duration time. Post-layout simulations indicate that pulse widths in the region 0.4–5.5 ns can be achieved through adjustment of VM. As such, the column is only occupied by one firing pixel for a short time, which allows photons from multiple pixels to be detected during the same cycle.

The collision detection bus was implemented in each column to share the address lines between 32 pixels, which enables event collision detection when two or more pixels fire simultaneously [15]. The diagram of the bus is shown in Figure 1, where 7-bit address lines ADDR [0:6] are connected to all the ALTDCs for address latching, and the TIMING line is shared by 4 TDCs for the conversion start triggering. Collision detection is achieved by implementing the pixel address in a winner-take-all (WTA) circuit such that each code has three ‘1’s and four ‘0’s, as is shown in Table 1. Since each pixel pulls down a different combination of address lines, if two or more pixels firing within the same pull down period, invalid addresses with more than three ‘1’s will be generated and distinguished.

### 2.3. Dynamic Reallocation of Time-to-Digital Converters

**Figure 3:**
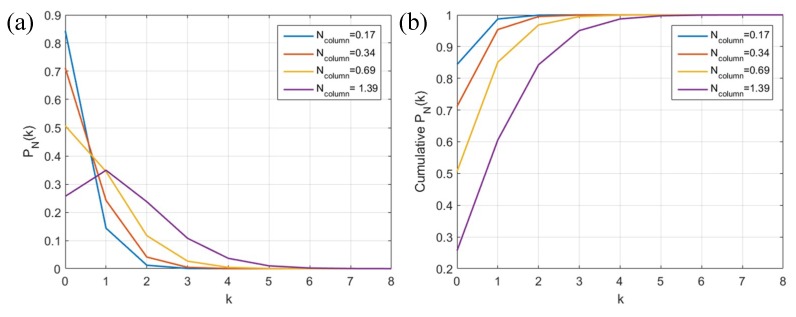
(**a**) Poisson distribution and (**b**) cumulative distribution of *P_N_*(*k*) at column activity of 0.17, 0.34, 0.69 and 1.39.

Since the detected photons have to be read off-chip for processing, the IO bandwidth determines the maximum photon throughput, so as the number of the photons that can be read out in each cycle. In this case, a per-pixel TDC architecture exhibits low efficiency in both power consumption and area occupancy, due to the sparse photon detection [13]. To improve the fill factor, instead of a per-pixel TDC architecture, a TDC sharing scheme is employed in this design. Nevertheless, the TDC bank is sized to achieve the same detection efficiency as that of per-pixel TDC architecture. Assuming the activity of each pixel is statistically independent, the light incident can be modeled with a Poisson distribution, given by Equation (1):

(1) $$P_{N}\left(k\right)=\;\frac{N_{column}^{k}*exp^{-N_{column}}}{k!}$$

where *P_N_*(*k*) is the probability of a number of *k* events detected in the column, while *N_column_* represents the average event rate of one column in one detection cycle. As each column is read out via a GPIO at 160 MHz and the event data length is 23 bits, thus a *N_column_* of 0.17, 0.34, 0.69 and 1.39 can be obtained at 40, 20, 10 and 5 MHz illumination frequency, respectively, which covers the complete TDC dynamic range. The probability distribution and cumulative distribution of *P_N_*(*k*) is shown in Figure 3. We can see that more than 95% of the events can be detected with only three TDCs per column in all the cases.

TDC sharing architectures have been implemented in some works [16], where one TDC is shared or multiplexed with a set of pixels. This limits the photon throughput due to the fact that one pixel firing will occupy the TDC and prevent other pixels in the same cluster to detect photons. In this paper, we propose a new TDC sharing architecture that dynamically reallocates 4 ALTDCs in one column for address latching and TOF measurement.

**Figure 4:**
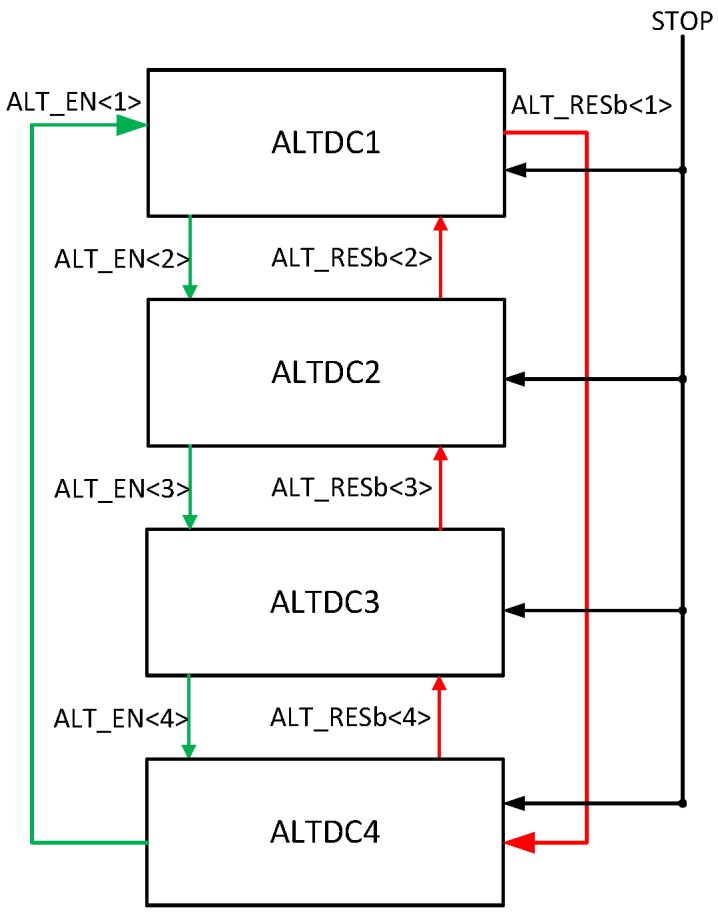
ALTDC daisy chain block diagram.

The block diagram of ALT is shown in Figure 4. The idea is to connect ALTDCs in a daisy chain approach and each ALTDC is enabled sequentially. At any time only one ALTDC is enabled for address latching and TOF detection, and each ALTDC is enabled by the previous block and then reset by subsequent block after data readout. As such, the ALTDCs are enabled and reset in sequence, driven by the column photon detection. TDC conversion is stopped by the signal STOP which is shared by the whole TDC array and synchronized with the laser clock. However, to prevent the entire ALTDC chain being reset by detection of four events in one cycle, there is always one ALTDC keeping inactive, which limits the maximum number of photons that can be detected in one cycle to be 3.

**Figure 5:**
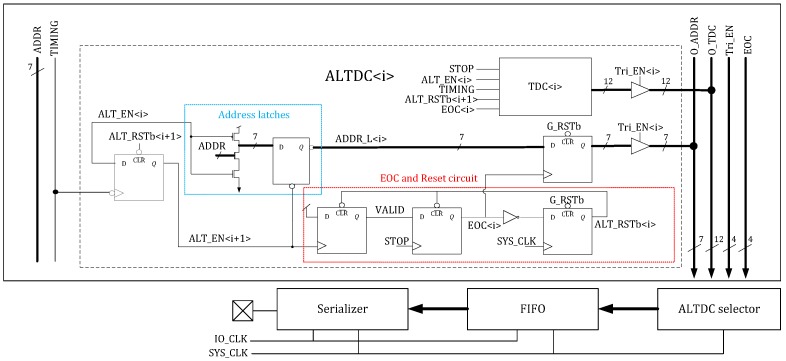
ALT schematic with the main functionality circuit and interface to the readout block.

A simplified ALTDC schematic is shown in Figure 5, which is enabled by ALT_EN<i-1>. Since the load capacitance in the ADDR and TIMING lines are different, which is mainly due to the different WTA circuit connection pattern of the pixels, the propagation delay on these lines would have a certain skew, so if the event addresses are latched synchronously at the rising edge of the TIMING signal, incorrect addresses could be captured due to the time skew and insufficient flip-flop setup time. Therefore, prior to the ADDR latching, addresses are firstly captured with a set of fast dynamic logic in synchronization with the rising edge of TIMING signal, where the correct addresses can be captured as long as the time skew is smaller than the pulse width. After that the dynamic logic outputs are latched at the falling edge of TIMING signal. With this method, the timing margin can be extended to the entire pulse width, which enables shorter pulses to be detected on the bus, thus reducing the bus dead time.

**Figure 6:**
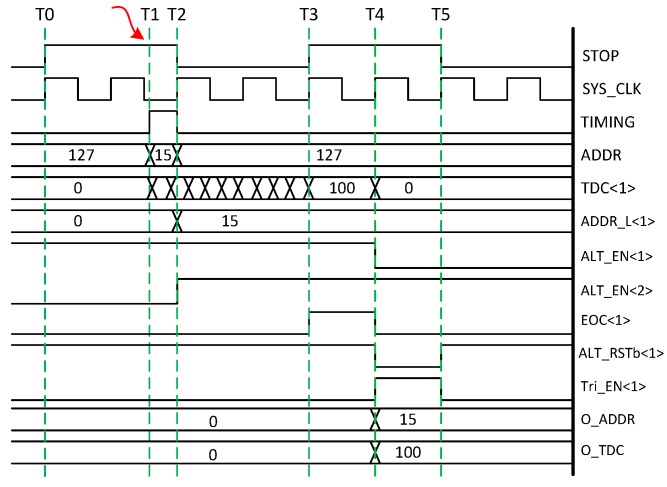
ALTDC timing diagram associated with photon detection by ALTDC<1>.

The ALTDC operation timing diagram, associated with photon detection by ALTDC<1> is shown in Figure 6, which is initialized after global reset, T0. When one pixel detects a photon, T1 in Figure 6, it generates a short pulse on TIMING line, the rising edge of the pulse then triggers the conversion of TDC<1>. At the same time, ALT_EN<1> deasserts the reset of the dynamic logic, which enables address capturing on ADDR bus. At the falling edge of TIMING, T2, ALT_EN<2> rises to logic high, which (1) enables ALTDC<2> for photon detection; (2) latches the address to ADDR_L<1>; (3) triggers VALID signal to begin event-driven readout process. At the end of the cycle, T3, TDC<1> is stopped by the rising edge of STOP, and signal EOC<1> is generated to indicate the availability of events, latching the address and TDC value into registers for readout. The readout block is synchronized with clock SYS_CLK, which is phase alighted with STOP to make sure the EOC signal can be sampled correctly. At the rising edge of SYS_CLK, T4, depending on EOC<1>, Tri_EN<1> is asserted to enable ALTDC<1> to read out through two shared tri-state buses, O_ADDR and O_TDC. Meanwhile, ALT_RSTb<1> is asserted to reset TDC<1>, EOC<1> and ALT_EN<1>. While they are released from reset at the next rising edge of SYS_CLK, T5, and the data on O_ADDR and O_TDC buses are registered into the FIFO for serial output.

The minimum time interval between photons that can be latched is limited by two factors: ADDR/TIMING pulse width and propagation delay of ALTDC chain. Because of the load capacitance mismatch between TIMING and ADDR buses, pulse skew and non-uniformity make it difficult to latch correct addresses when using short pulses. A minimum photon interval of 1.2 ns is obtained from post-layout simulation. To improve the readout efficiency, event-driven readout method was implemented, where only the ALTDCs that detect photons will be read out. No power is dissipated communicating null events, where no photon impinges, which is typically the case for TDC per-pixel architectures [11]. On the other hand, this approach shows excellent scalability that any length of the daisy chain can be built by simply cascading ALTDCs, without changing any signals except Tri_EN and EOC for the readout, which reduces the complexity of building larger arrays in bus sharing architectures.

**Figure 7:**
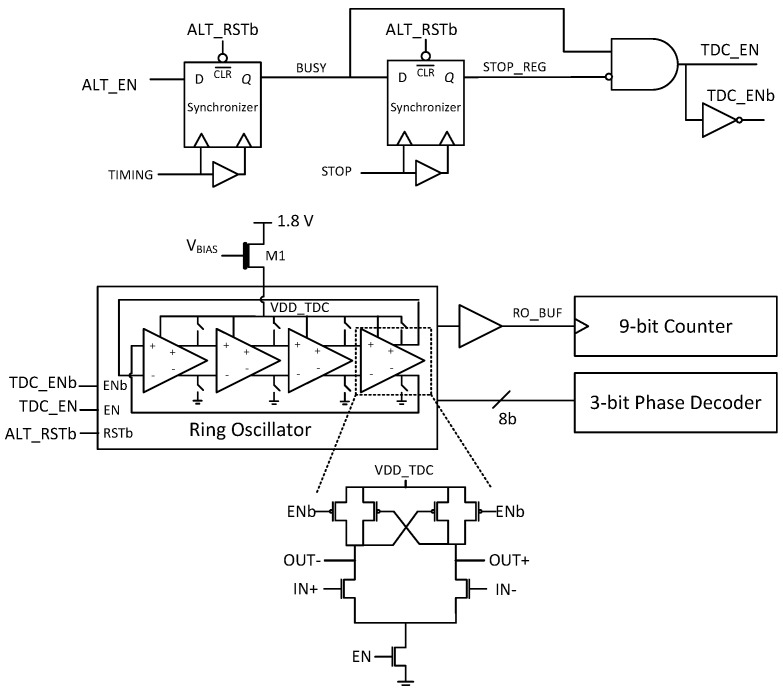
TDC schematic based on a four-stage differential ring oscillator.

The TDC is based on a differential four stage ring oscillator (RO), shown in Figure 7. Synchronizers were designed to reduce the metastability when the asynchronous signals TIMING and BUSY switch from ‘0’ to ‘1’ [15]. A thick oxide NMOS transistor M1 is used to regulate the voltage supply for the ring-oscillator to mitigate against frequency variations due to IR drops in the ALTDC array. A 9-bit counter connected to the RO clock operates at 2.5 GHz, which provides coarse resolution of 400 ps. A phase discriminator resolves the 8-bit thermometer coded phases and converts them to a 3-bit binary code, leading to a fine resolution of 50 ps. The 128 column TDCs sharing one common control voltage VBIAS are externally biased, where process-voltage-temperature (PVT) compensation can be implemented off chip via an on-chip replica RO.

### 2.4. Chip Realization

**Figure 8:**
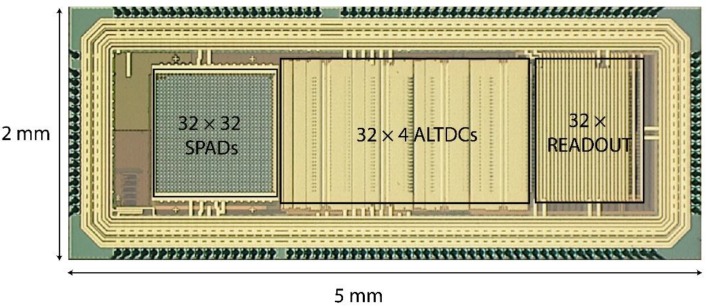
Chip microphotograph.

The sensor was fabricated in a 180 nm CMOS technology, and a microphotograph of the chip with dimension of 5 mm × 2 mm is shown in Figure 8. An array of 32 × 32 pixels was implemented, where 4 pixels are not connected to the main array and only used for SPAD characterization purposes.

## 3. Measurement Results

### 3.1. Chip Pixel Characterization

**Figure 9:**
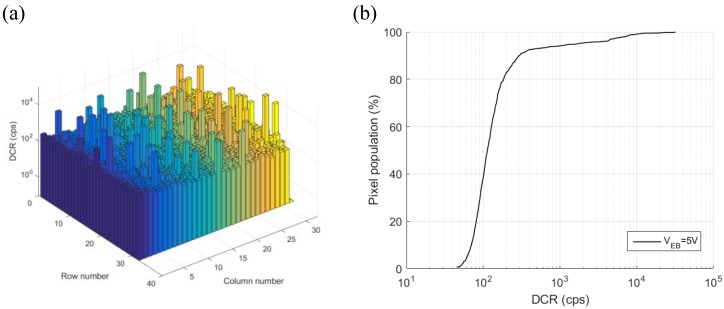
(**a**) DCR map and (**b**) DCR cumulative proportion of the whole array with 5 V excess bias voltage at 20 °C.

The SPAD in this design stands as one of the best CMOS SPADs in terms of DCR, yield and PDP so far reported [19,20,21,22,23]. The breakdown voltage was measured at 22 V. DCR measurement at 5 V excess bias voltage of the whole array is shown in Figure 9, where the median value is 113 cps with an active area of 231 μm^2^, which corresponds to a DCR density of 0.49 cps/μm^2^ at the temperature of 20 °C. Furthermore, high DCR uniformity is achieved, where more than 94% of the SPADs have a DCR less than 1 kcps. No obvious afterpulsing was observed with 25 ns dead time at 5 V excess bias voltage. This is in agreement with [17], where the afterpulsing of the same device was measured at 0.08% at an excess voltage of 4 V. Notably, the result in [17] was also achieved without an integrated quenching circuit, this increases the capacitance at the SPAD anode and degrades the afterpulsing performance due to increased carrier flow during an avalanche [24].

**Figure 10:**
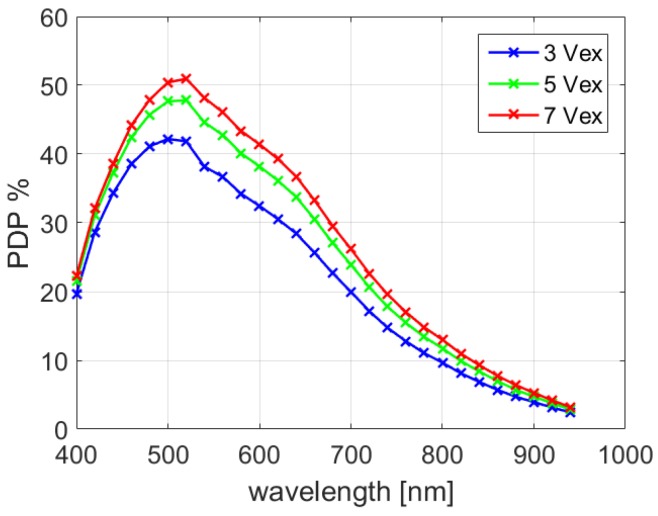
PDP measurement at excess bias voltage of 3 V, 5 V and 7 V.

The photon detection probability (PDP) characterization is shown in Figure 10, where a peak value of 47.8% was achieved at a wavelength of 520 nm with 5 V excess bias. More importantly, a high PDP of 8.4%, 4.7% and 2.8% was achieved at 840, 900 and 940 nm respectively, which provides more flexibility for 3D imaging at near infrared wavelengths. More than 50% peak PDP was achieved at 7 V excess bias voltage, while the reliability of the quenching circuit is not guaranteed. The full-width-at-half-maximum (FWHM) jitter was measured with a 637 nm laser, which reveals a jitter of 106 ps at 5 V excess bias. Since the jitter of the laser is 40 ps, the pixel jitter, including SPAD, quenching circuit, and IO buffer, can be extracted to be 98 ps.

### 3.2. TDC Characterization

In order to characterize the TDCs, a code density test was used. SPAD pixels are employed to generate uncorrelated START signals to trigger the conversion of the TDCs. The STOP signal (Figure 5), which is generated in the FPGA and fed to each TDC through a latency balanced clock tree, is used to stop the conversion. If one or less event is detected during the conversion period, the distribution of times of arrival should be uniformly distributed. The simplest way of generating uncorrelated signals with SPADs is to detect the dark count events. By acquiring enough events, e.g., >10^4^ events per bin, the TDC resolution (1 LSB), differential non-linearity (DNL) and integral non-linearity (INL) can be calculated based on the code histogram.

**Figure 11:**
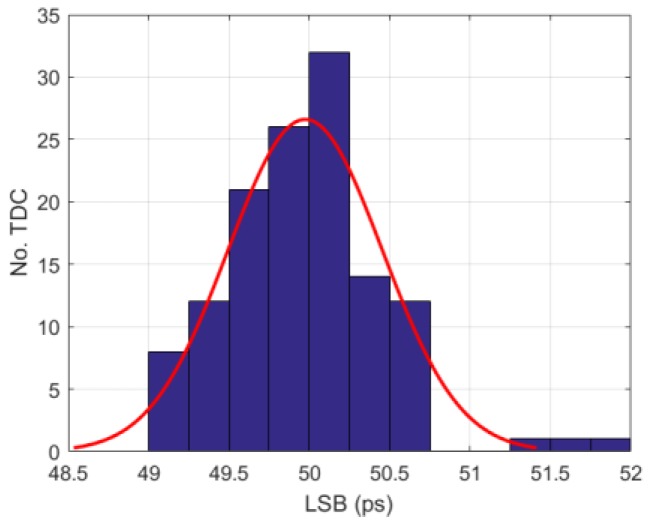
LSB distribution of the 128 TDCs shows a standard deviation of 0.48 ps.

**Figure 12:**
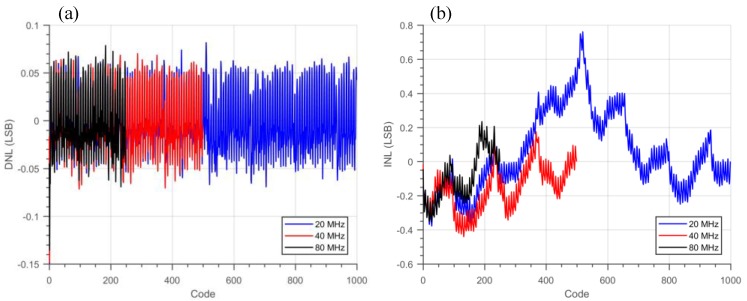
TDC (**a**) DNL and (**b**) INL measurement with different STOP frequency.

**Figure 13:**
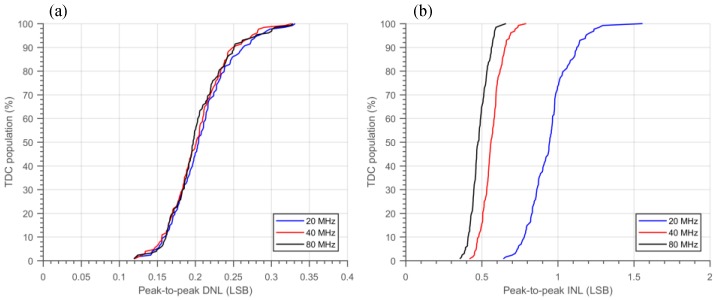
Peak-to-peak (**a**) DNL and (**b**) INL cumulative distribution with different STOP frequency.

The nominal bin size or resolution (LSB) of the TDC is 50 ps, where the RO operates at 2.5 GHz. The LSB variation of all the 128 TDCs is presented in Figure 11; a standard deviation across the whole array of 0.48 ps is achieved. The DNL and INL measurement results obtained from the code density test is shown in Figure 12, where −0.07/+0.08 LSB DNL and −0.38/+0.75 LSB INL were achieved with a 20 MHz STOP signal. From the measurement, a periodic DNL/INL nonlinearity component can be observed; this behavior is due to a weak coupling of the SYS_CLK to the RO bias voltage. Figure 13 shows the peak-to-peak (p2p) DNL and INL cumulative distribution of all the TDCs. As expected, the p2p INL is proportional to the TDC conversion time, since more noise is coupled and accumulated. Even so, a median p2p DNL and INL of 0.21 LSB and 0.92 LSB were achieved at 20 MHz STOP signal, which shows high homogeneity across the image sensor despite the fact that no PVT compensation was applied to the TDCs.

**Figure 14:**
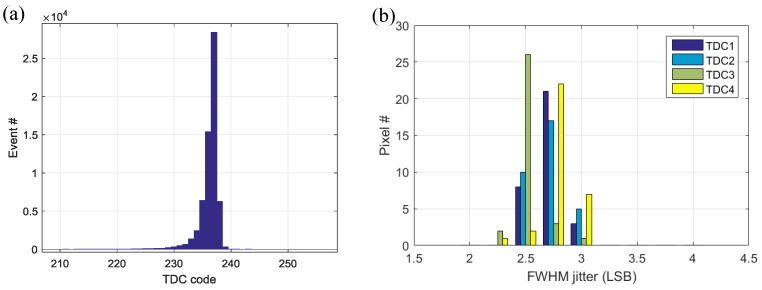
(**a**) Single shot SPAD-TDC timing jitter measurement with a minimum FWHM of 2.28 LSB (**b**) jitter distribution of all the pixels at each TDC measurement, leading to the average and standard deviation of 2.68 LSB and 0.15 LSB, respectively.

The SPAD-TDC single-shot timing characterization was obtained by illuminating the sensor with a pulsed laser at wavelength of 637 nm. A 5 V excess bias voltage was applied, achieving a minimum FWHM jitter of 2.28 LSB (114 ps), as is shown in Figure 14a. The jitter distribution of the 32 pixels with respect to each of the four TDCs is shown in Figure 14b, where good uniformity is achieved with the average and standard deviation of 2.68 LSB (134 ps) and 0.15 LSB (7.5 ps) respectively. No obvious degradation of the jitter is observed during the signal propagation through the complete length of the bus and ALTDC daisy chain. As is discussed in Section 3.1, the jitter of the laser and pixel is 40 and 98 ps, respectively; the TDC quantization error at FWHM is 34 ps. Therefore, we can obtain the average jitter from the entire collision detection bus and ALTDC daisy chain is only 75 ps. It implies this architecture could be scaled to build larger pixel arrays.

### 3.3. Flash 3D Imaging

**Figure 15:**
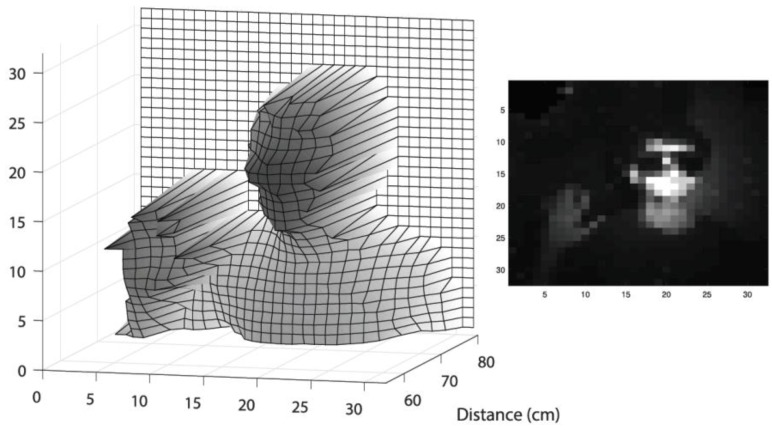
Flash 3D imaging of a human subject at distance of 0.7 m with 2D intensity image inset.

To validate the sensor, a flash 3D imaging measurement was performed, where a target was illuminated with a diffused laser and the reflected light collected on a per-pixel basis. An objective was placed in front of the sensor, enabling a field-of-view (FOV) of 40 degree × 40 degree. A Xilinx Kintex-7 based FPGA evaluation board (XEM7360, Opal Kelly, Portland, OR, USA) was used to read out the TOF events, then transmit to the computer via a USB 3 interface. Finally, a 3D image can be constructed by histogramming the TOF data of each pixel. TDC calibration was applied for LSB variations among different TDCs, as well as time offset due to the skew of STOP clock. As is shown in Figure 15, a 3D image was obtained, where a person wearing a laser protection glass and with the right hand raised standing at a distance of 0.7 m away from the sensor. The target was illuminated at a wavelength of 637 nm. Due to the limited laser power, the measurement was performed at dark conditions and with an exposure time of a few seconds. Millimetric detail can be observed thanks to the low timing jitter of the system and high single-to-background ratio (SBR).

### 3.4. Scanning LiDAR Experiment

**Figure 16:**
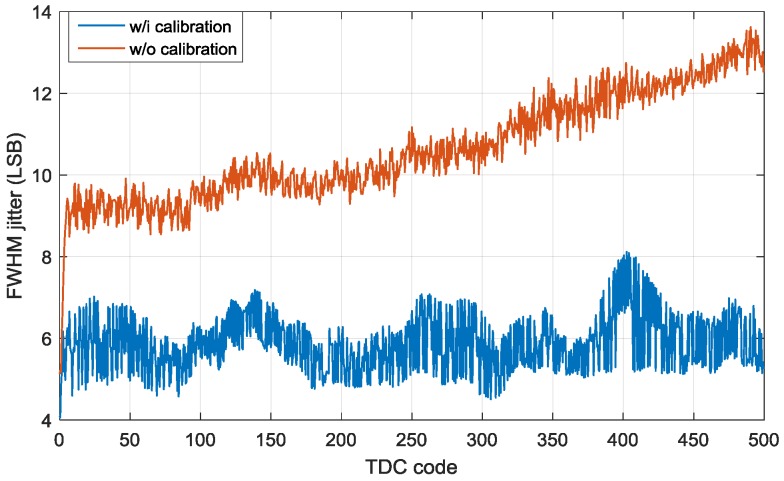
Jitter measurement before and after calibration, where the average jitter is reduced from 10.63 LSB to 5.87 LSB.

In order to extend the image resolution, the sensor was demonstrated in a scanning LiDAR system. To perform scan imaging, the entire pixel array was used as a single detection component, where the mismatching between TDCs is accumulated with time. To improve the accuracy of the measurement, calibration has to be applied to each TDC and SPAD. The single shot timing response of the whole array, Figure 16, was acquired by electrically sweeping the STOP signal at a phase shift step of 25 ps.

As expected, the jitter is proportional to the TDC value, as more nonlinearity error is accumulated with distance. To improve the linearity, calibration was performed to two major parameters, comprising the TDC LSB variation and signal skew. Prior to the calibration, one SPAD in the center of the array (row 16 and column 16) and the first TDC in the column are used as a reference for the calibration alignment of all the pixels and TDCs. For the TDC LSB variation, since the LSB of every TDC is characterized in Figure 11, the TDC code offset can be calculated with respect to the reference TDC. The signal skew, including the delay in the pixel circuit, TIMING and STOP signals, is calibrated by illuminating the sensor with a laser and the delay offset of each SPAD with respect to each TDC is measured and stored in a look-up table for calibration. As is shown in Figure 16, after calibration the FWHM jitter is stabilized and reduced from 10.63 LSB to 5.87 LSB in average. However, the average jitter of a single pixel from a single TDC is 2.68 LSB, which is smaller than that of the system jitter of 5.87 LSB. Two main factors contribute to the calibration degradation. The first one is the calibration quantization error. The calibration coefficient is stored in look-up tables in the FPGA for real-time calibration and imaging. However, to reduce the complexity of the firmware, the value of the coefficient is rounded to the nearest integers, which introduces quantization error and reduces the calibration accuracy. Another reason is the temperature and voltage dependence of the calibration. Since all the ROs operate in open loop, the frequency is varying over temperature and voltage, leading to a TDC linearity, which is difficult to calibrate with a constant coefficient. A similar situation also occurs to the propagation delay of SPAD output, TIMING and STOP signals. Nevertheless, the oscillation of ROs could be stabilized by locking the frequency with an external phase-locked loop, which improves the frequency tolerance to temperature and voltage variation. While for the skew calibration, different measurement could be performed at different operating conditions to retrieve temperature-voltage dependent look-up tables.

**Figure 17:**
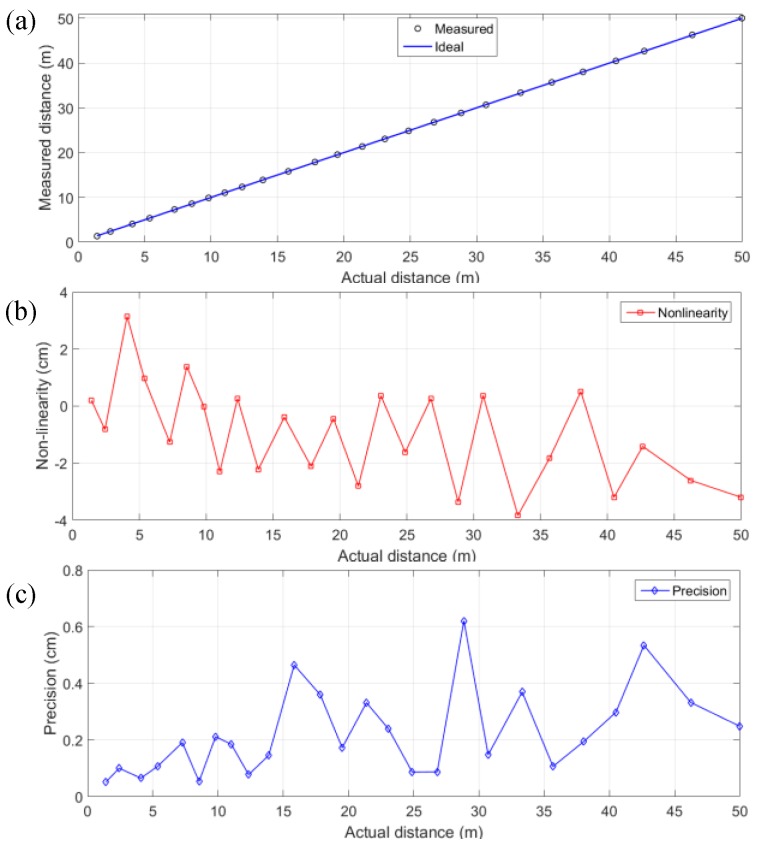
(**a**) Measured distance up to 50 m as a function of the actual distance; (**b**) The maximum non-linearity and (**c**) worst-case precision were achieved at 6.9 cm and 0.62 cm respectively.

Single-point telemetry, shown in Figure 17, was performed with the same laser at 40 MHz repetition rate, 2 mW average power, and 40 ps pulse width. Even though the unambiguous range with a 40 MHz laser is 3.75 m, larger range can be characterized by exploiting the prior knowledge of the distance offset. In this way, the linearity of the system was characterized and shown in Figure 17. A 60% reflectivity target was measured up to 50 m, where each distance was measured for 10 repeated times in dark conditions, achieving a maximum non-linearity and worst-case precision (σ) of 6.9 cm and 0.62 cm respectively, over the entire range. Instead of controlling the frame time, a constant amount of 50 k photons were collected in every measurement, which gives a high SBR and detection reliability. In this case, the object signal can be distinguished, even though the distance is 50 m and the laser peak power is only 0.5 W. However, if the background light is high and the frame time is limited, less signal photons will be acquired and the performance will be degraded with distance.

**Figure 18:**
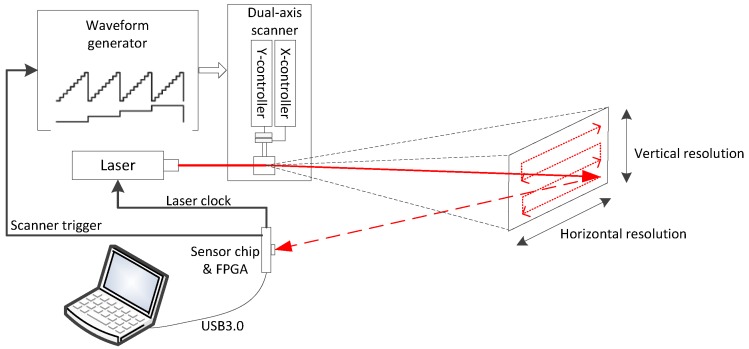
Block diagram of the LiDAR system.

Based on the sensor, a scanning LiDAR system comprising a dual-axis galvanometer scanner (GVS012, Thorlabs, Newton, NJ, USA), an arbitrary waveform generator (AWG, 33600A, Keysight, Santa Rosa, CA, USA), and a 637 nm laser source was built, as is shown in Figure 18. Two channels of step signals are generated by the AWG to drive the scanner to perform configurable raster scan on the target.

**Figure 19:**
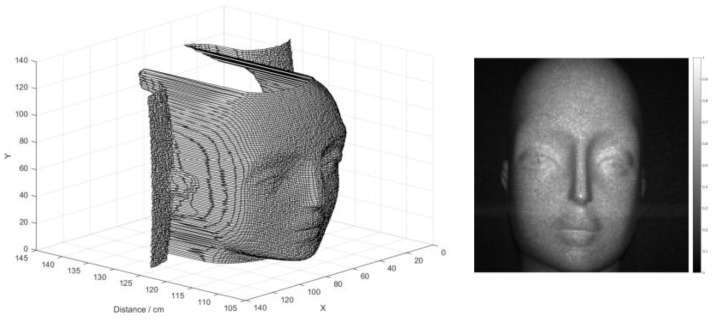
Scan imaging of a mannequin at distance of 1.3 m with a resolution of 128 × 128, where both the depth and intensity images were obtained at the same time.

The scan experiment was performed in dark conditions, where a mannequin was placed 1.3 m away from the sensor with curved background. The facial image of the mannequin was obtained in Figure 19, where both depth and intensity images were acquired with a resolution of 128 × 128 at the same time. The scanner was operated at a low frequency of 1 KHz, which ensures more than 10 K photons being detected at each scanning point, thus enabling a high SBR. The distance of each point was calculated by averaging the bins around the peak of the histogram. Millimetric depth resolution was achieved, where details of the face can be clearly recognized, which proves the high linearity of the scanning system.

**Figure 20:**
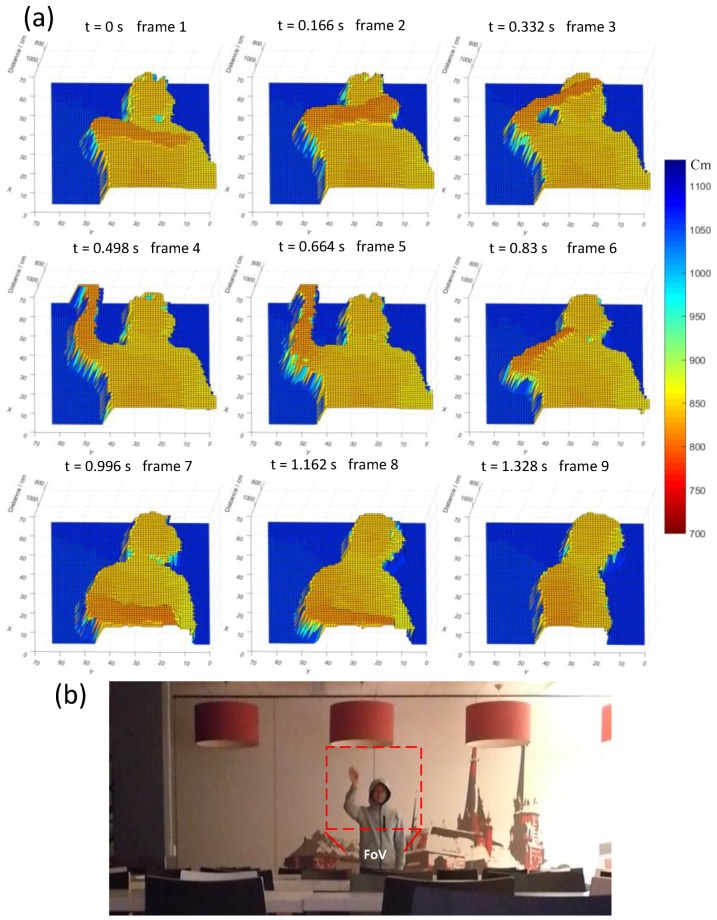
(**a**) Nine consecutive frames were recorded at 6 frames/s with resolution of 64 × 64 at 10 m, where a human subject was waving his right hand and turning around; (**b**) image captured with a commercial camera.

Furthermore, real-time imaging was carried out with 50 lux of background light at a resolution of 64 × 64, where the scanner was operated at a frequency of 24.5 KHz. As is shown in Figure 20, a human subject (reflectivity of about 40%) standing 10 m away to the sensor, waving the right hand and turning around, was recorded at 6 frames/s. The FOV was adjusted to be 5 degree × 5 degree, which gives a fine angular resolution of 0.078 degree in both X and Y directions, corresponding to a scanning step of 1.36 cm per point. To improve the SBR, a bandpass optical filter with FWHM of 10 nm was used to suppress the background light. Thanks to the high PDP and photon throughput, sharp images were recorded with an average and peak laser power as low as 2 mW and 500 mW, respectively. Since a low power laser and visible wavelength were employed in the experiment, we believe the ranging performance can be significantly improved by using a high power near-infrared laser, without affecting other aspects of the system.

Table 2 summarizes the results of the whole system, including the chip characteristics, distance measurement and the LiDAR system performance. The total power consumption, which is strongly dependent on the operating environment, has been measured at 0.31 W where the photon throughput is about 35.5 Mcps. The ALTDC array, readout logic, IO interface, pixel array and debugging circuits contributed 30%, 28%, 27%, 11% and 4% respectively.

## 4. Proposed Background Light Suppression Architecture

In order to improve the tolerance to the background light in SPAD sensors, coincidence photon detection has been applied, whereas only events with more than one photon detected in a coincidence time window are processed by the sensor. In [6,7], referred to as method 1, the authors implemented multiple stages of adders to quantify coincidence photons. In this method, one continuously counts the number of events detected by a set of SPADs in a predefined time window. By compensating the propagation delay between the signal outputs and carrier outputs of the adders, TDCs can only be triggered by the second photon of the coincidence event. While for a number of ***N*** SPADs, the same number of bits has to be summed up, which requires a large number of adders thus limiting the suitability of the approach in large arrays. In [5], SPADs were combined onto a single output via independent monostables and a balanced OR-tree. In this method, referred to as method 2, the output of the OR-tree drives a series of shift registers to count the events and validate coincidence detection. Since the silicon area of the OR-tree is much smaller than that of the adder-tree, a 64 × 64 pixel array could be implemented. However, when multiple photons are detected at a time distance shorter than the pulse generated by the monostable, the OR-tree can only output one pulse, which results in events missed and reduced SBR. Another drawback is that the TDCs are always triggered by the first event, while in case of uncorrelated photons the TDCs will be reset after the coincidence window. Therefore, a high TDC activity and power consumption can be expect with strong background light.

Even though background light suppression is not explicitly implemented in this design, with the intrinsic capability of coincidence photon detection, the collision detection bus can be seen as such, since the coincidence window begins with the first photon detected and ends a user-determined time delay later. This implicit coincidence window is an effective approach to background suppression methods, since coincident events (light source) can be recognized within the window and signals (noise and background) can be easily suppressed outside it.

**Figure 21:**
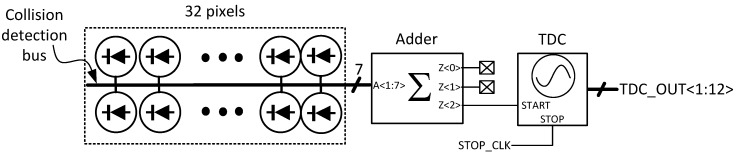
Proposed sensor architecture with coincidence event detection among 32 pixels, based on collision detection bus.

The proposed sensor architecture is shown in Figure 21, where a group of 32 pixels are employed for coincidence detection. As is discussed in Section 2.2, collision events will generate an address output with more than three non-zero bits. Therefore, when coincidence events are detected, the most-significant bit (MSB) of the adder, Z<2>, will rise to high, which can be directly used for the triggering of the TDC.

In comparison with method 1 and 2, instead of 32 bits, only seven bits have to be processed, so a much smaller coincidence detection circuitry can be constructed. More specifically, to perform coincidence detection with 32 pixels, an adder tree with 13 full-adders, four half-adders, one AND gate and one 18-input OR gate is required in method 1, while an OR-tree with 31 NAND/NOR gates for method 2. Instead, the proposed approach only needs three full-adders and four half-adders. On the other hand, since it is based on an adder, the event-miss problem in method 2 would not happen in this approach. Furthermore, in comparison with method 2, since the TDC can only be triggered by the coincidence events with the output of Z<2>, low TDC activity can be achieved thus leading to low power consumption. In comparison to the current sensor architecture, only a minor modification with the implementation of the adders is required, which prevents the features of the pixel array from being affected. Therefore, with the proposed approach, a SPAD sensor with a large pixel array, higher fill factor and high background light suppression is expected.

## 5. Conclusions

In this work, we presented a 32 × 32 SPAD imager, fabricated in a 180 nm CMOS technology, where each 32 pixels in one column are connected to a collision detection bus. With the bus-sharing scheme, a fill factor of 28% and a pixel pitch of 28.5 μm were achieved. To improve the photon throughput, a scalable ALTDC mechanism was implemented to dynamically reallocate TDCs for TOF events detection. This enables the same photon throughput as that of per-pixel TDC architectures. The events are read off-chip in an event-driven readout method with high energy efficiency, where 32 channels are employed operating at a bandwidth of 5.12 Gbps, which enables a maximum throughput of 222 Mcps and 465 Mcps in PT and PC mode, respectively. The SPAD exhibits 47.8% PDP at 520 nm, 113 cps median DCR, 106 ps FWHM jitter and negligible afterpulsing was characterized at an excess bias of 5 V. Ranging measurement at a distance of 50 m achieved 6.9 cm non-linearity (0.14% accuracy) and 0.62 cm precision (σ = 0.01%). Based on the sensor, a scanning LiDAR system achieving depth imaging up to 10 m at 6 frames/s with a resolution of 64 × 64 pixels was demonstrated with 50 lux of background light. The average and peak illumination power was as low as 2 mW and 500 mW respectively. This sensor provides flexibility for applications in which low light imaging and high timing resolution are required, such as quantum imaging, biological imaging, as well as indoors flash and scanning LiDAR. To improve the background light suppression, a new sensor architecture based on the concept of collision detection bus is proposed. Compared to other methods in literature, the proposed method has the benefit of reduced coincidence detection circuitry area and low TDC power consumption, which provides an approach of designing SPAD sensors with a large pixel array and high fill factor for TOF imaging applications in high background light environment, such as automotive LiDAR.

## Figures and Tables

**Table 1:** Address code table for 32 pixels.

SPAD	ADDR	SPAD	ADDR	SPAD	ADDR	SPAD	ADDR
0	1110000	8	1000011	16	0011001	24	0001110
1	1100100	9	1000101	17	0011010	25	0101100
2	1100001	10	1000110	18	0010011	26	0101001
3	1100010	11	1010100	19	0010101	27	0101010
4	1101000	12	1010001	20	0010110	28	0100011
5	1001100	12	1010010	21	0000111	29	0100101
6	1001001	14	1011000	22	0001011	30	0100110
7	1001010	15	0011100	23	0001101	31	0110100

**Table 2:** Performance summary of the sensor and LiDAR system

Parameter	Value	Unit
Chip characteristics		
Array resolution	32 × 32	
Technology	180 nm CMOS	
Chip size	5 × 2	mm^2^
Pixel pitch	28.5	μm
Pixel fill-factor	28	%
SPAD break down voltage	22	V
SPAD median DCR	113 (Vex = 5 V, 20 °C)	cps
SPAD jitter	106 (Vex = 5 V)	ps
SPAD PDP	47.8 (Vex = 5 V @520 nm)	%
TDC LSB	50	ps
TDC resolution	12	bit
No. TDC	128	
TDC area	4200	μm^2^
Readout bandwidth	5.12	Gbps
Maximum photon throughput	222 (PT mode)	Mcps
	465 (PC mode)	Mcps
Distance measurement		
Measurement range	50	m
Non-linearity (Accuracy)	6.9 (0.14%)	cm
Precision (σ) (Repeatability)	0.62 (0.01%)	cm
LiDAR experiment		
Illumination wavelength	637	nm
Illumination power	2 (average)	mW
	500 (peak)	mW
Frame rate	6	fps
Image resolution	64 × 64	
Field of view (H × V)	5 × 5	degree
Target reflectivity	40	%
Distance range (LiDAR)	10	m
Background light	50	lux
Chip power consumption	0.31 (@ 35.5 Mcps photon throughput)	W
